# The thrombospondin-1 receptor CD36 is an important mediator of ovarian angiogenesis and folliculogenesis

**DOI:** 10.1186/1477-7827-12-21

**Published:** 2014-03-14

**Authors:** Kata Osz, Michelle Ross, Jim Petrik

**Affiliations:** 1 Department of Biomedical Sciences, University of Guelph, Guelph, ON N1G 2W1, Canada

**Keywords:** Ovary, CD36, Thrombospondin, Folliculogenesis, Angiogenesis

## Abstract

**Background:**

Ovarian angiogenesis is a complex process that is regulated by a balance between pro- and anti-angiogenic factors. Physiological processes within the ovary, such as folliculogenesis, ovulation, and luteal formation are dependent upon adequate vascularization and anything that disrupts normal angiogenic processes may result in ovarian dysfunction, and possibly infertility. The objective of this study was to evaluate the role of the thrombospondin-1 (TSP-1) receptor CD36 in mediating ovarian angiogenesis and regulating ovarian function.

**Methods:**

The role of CD36 was evaluated in granulosa cells in vitro and ovarian morphology and protein expression were determined in wild type and CD36 null mice.

**Results:**

In vitro, CD36 inhibition increased granulosa cell proliferation and decreased apoptosis. Granulosa cells in which CD36 was knocked down also exhibited an increase in expression of survival and angiogenic proteins. Ovaries from CD36 null mice were hypervascularized, with increased expression of pro-angiogenic vascular endothelial growth factor (VEGF) and its receptor VEGFR-2. Ovaries from CD36 null mice contained an increase in the numbers of pre-ovulatory follicles and decreased numbers of corpora lutea. CD36 null mice also had fewer number of offspring compared to wild type controls.

**Conclusions:**

The results from this study demonstrate that CD36 is integral to the regulation of ovarian angiogenesis by TSP-1 and the expression of these family members may be useful in the control of ovarian vascular disorders.

## Background

Ovarian folliculogenesis, ovulation, and formation of the corpus luteum require complex and coordinated interaction of numerous autocrine, paracrine, and endocrine factors to regulate important physiologic processes such as angiogenesis. Angiogenesis is the formation of new blood vessels from pre-existing vasculature. In the adult, angiogenesis is generally quiescent except for the vascularization required for wound healing. However, the female reproductive tract, including the ovaries, and uterus are unique as they are adult tissues that undergo cyclic angiogenic processes to facilitate processes important to reproduction. An important regulator of ovarian angiogenesis is vascular endothelial growth factor (VEGF), which stimulates endothelial proliferation, migration, and survival, and we have shown that through interaction with its receptor VEGFR2, it is an important survival factor for extravascular ovarian cells [1].

Thrombospondin-1 (TSP-1) is a large (450 kDa) multimodular glycoprotein encoded by the *THSB1* gene that was originally identified as a major component of platelet α-granules [2,3]. Since then, TSP-1 has been shown to be an important matricellular protein that mediates cell signaling, wound healing, cell adhesion, and angiogenesis [4,5]. Of TSP-1′s many biological functions, likely the most notable effect is the inhibition of angiogenesis [6]. The effects of TSP-1 in tumorigenesis have been well-studied. Reduced expression of TSP-1 is usually associated with aggressive angiogenesis and enhanced tumour formation [7], while TSP-1 over-expression generally results in inhibited tumour formation and reduced tumour vessel density [8]. During tumour development, TSP-1 is typically inversely associated with pro-angiogenic markers such as vascular endothelial growth factor (VEGF) where tumours with a higher TSP-1:VEGF ratio are typically slower growing, while tumours that favor VEGF expression are aggressive and have a higher rate of malignancy [9,10].

CD36 is a multi-ligand glycoprotein receptor, although it is best known for binding TSP-1. CD36 is known to act as a transporter and sensor of free fatty acids [11,12] and a scavenger receptor that binds a number of factors and participates in the internalization of cells, pathogens, and various lipoproteins [13]. CD36 is an 88-kDa integral plasma membrane protein and is expressed by a variety of cells including platelets, erythrocytes, endothelial cells, monocytes, granulosa, theca, and tumour cells [14-16]. Likely one of the most well-established functions of CD36 is the inhibition of angiogenesis following binding to the Type I repeats (TSR) of TSP-1 and −2 [17,18]. Following binding of TSR, CD36 initiates anti-angiogenic signals through the induction of endothelial cell apoptosis [19,20]. TSP-1 stimulates CD36 to recruit non-receptor protein kinases fyn, lyn and yes to the CD36 complex [21]. This recruitment results in the activation of the kinase and initiation of the p38 mitogen activated protein kinase (MAPK) pathway, initiation of caspase-3-like effectors, and ultimately apoptotic cell death [14].

TSP-1 and CD36 are coordinately expressed in granulosa and theca cells of rodent [16] and bovine [15] ovaries. Expression of the ligand and receptor vary throughout the reproductive cycle and are regulated at least in part by gonadotropins [16]. Cyclical expression of TSP-1 is associated with changes in ovarian angiogenesis, where reduced expression of the protein accompanies increased perafollicular and luteal angiogenesis during period of follicular and luteal development [16].

We have shown that TSP-1 Null mice are subfertile and have altered ovarian morphology highlighted by increased vascularization and disrupted follicle dynamics, compared with wild-type controls [22]. TSP-1 and its mimetic peptides have been shown to reduce VEGF expression, inhibit ovarian angiogenesis, and induce follicle atresia [23,24]. These findings led us to believe that TSP-1 and pro-angiogenic VEGF had reciprocal inhibitory influences in the ovary and we demonstrated that TSP-1 bound VEGF, resulting in its internalization and degradation through the low density lipoprotein receptor related protein (LRP)-1. However, the extent of the ovarian morphological and functional alterations in the TSP-1 null mice could not be accounted for exclusively by a direct interaction between TSP-1 and VEGF. We anticipated that in addition to VEGF-mediated effects, CD36 signaling may have specific influences on ovarian function. In ovarian tumor development, we demonstrated that treatment with TSP-1 mimetic peptides inhibited tumor formation, and that this effect was abrogated when CD36 expression was knocked down [25]. Based on the coordinated expression of TSP-1 and CD36 during ovarian follicle development, and the results from our work in the ovarian cancer model, we hypothesized that CD36 is an important mediator in the regulation of ovarian cell function and folliculogenesis.

## Methods

### Cell lines and tissue collection

Spontaneously immortalized rat granulosa cells (SIGC) were generously provided by Dr. Robert Burghardt (Texas A&M University, College Station, TX) and were cultured in DMEM/F12 (Gibco BRL), supplemented with 10% FBS, and 1% Antibiotic/Antimycotic (Gibco BRL). The SIGCs are derived from primary rat ovarian granulosa cell cultures and grow in culture without undergoing luteinization [26]. We have previously shown that these cells express the CD36 receptor [16]. CD36-null mice and wild type (WT) C57BL-6 littermates were a generous gift from Dr. Arend Bonen, Department of Health and Human Nutrition, University of Guelph, Guelph, ON. All animal work was carried out in compliance with guidelines established by the Canadian Council on Animal Care. For in vivo tissue collection, approximately 30-week old littermate WT and CD36^-\-^ mice (n = 8/group) were injected intraperitoneally with 2.5 IU PMSG (Sigma) to initiate a synchronous follicular wave. 48 hr after injection, mice were euthanized via CO_2_ asphyxiation and ovaries were collected at the late antral/preovulatory phase of the cycle and either flash frozen for protein collection, or fixed overnight in 10% neutral buffered formalin.

### CD36 knockdown in SIGC

*miRNA construct:* The following complementary single stranded oligonucleotides were synthesized by Sigma-Aldrich: Rmi615640_top_cd36 - TGCTGTTCCTTGGCTAAATAACGAACGTTTTGGCCACTGACTGACGTTCGTTATAGCCAAGGAA.

Rmi615640_bot_Cd36 - CCTGTTCCTTGGCTATAACGAACGTCAGTCAGTGGCCAAAACGTTCGTTATTTAGCAAGGAAC.

Oligonucleotide strands were annealed and ligated into pcDNA 6.2-GW/EmGFP-miR vector (Life Technologies) then transformed into TOP10 cells (Life Technologies) according to the manufacturer’s instructions. In addition to the CD36 knockdown oligonucleotides, scrambled sequence oligos with similar G/C content (Life Technologies) were also used as recommended.

**Figure 1:**
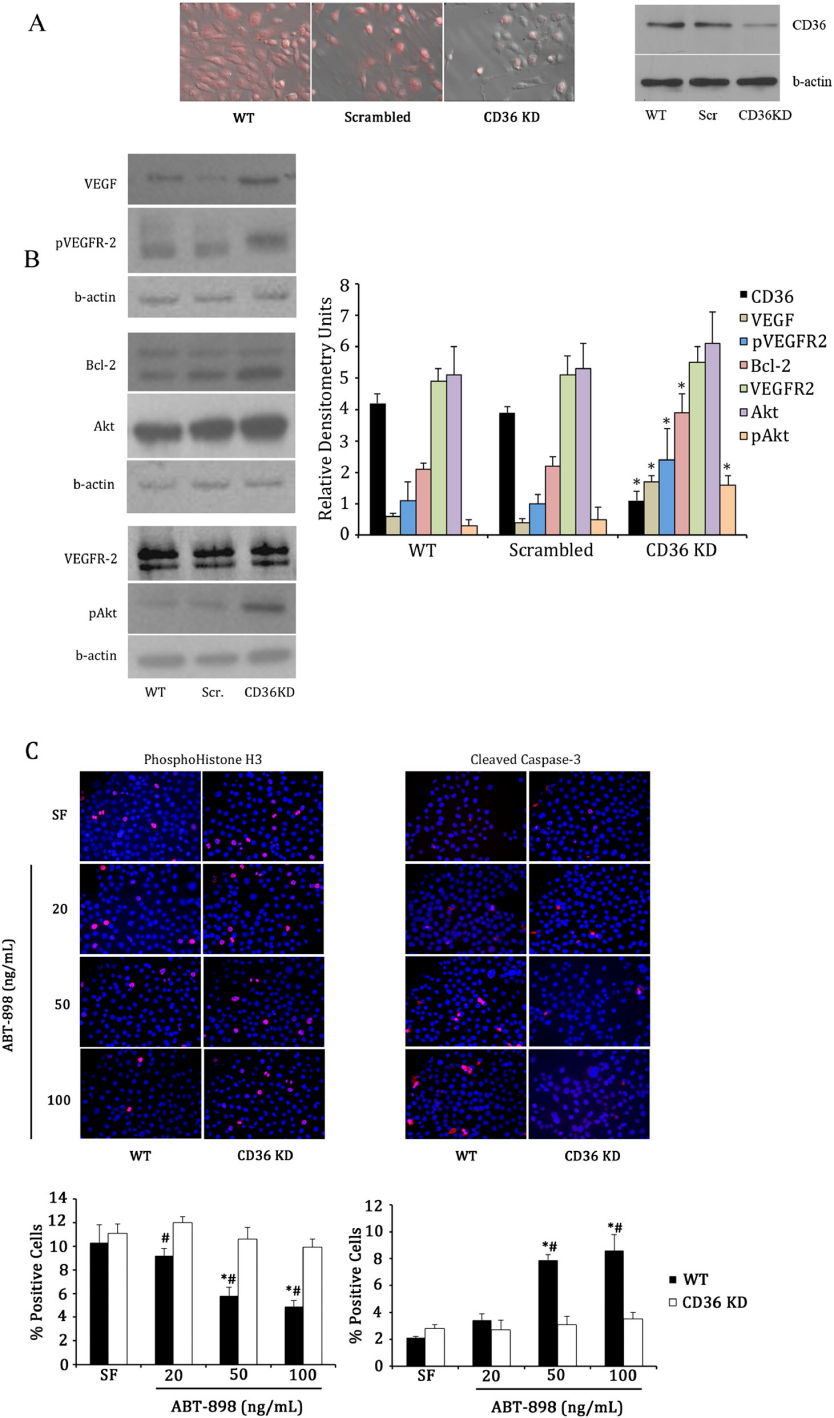
**Expression of survival factors and the effects of the TSP-1 mimetic peptide ABT-898 in vitro. A**. siRNA inhibition reduced expression of CD36 in spontaneously immortalized rat granulosa cells (SIGC) in vitro, as observed using brightfield/darkfield immunofluorescence overlay and Western blotting. **B**. Western blotting revealed that CD36 knockdown cells had increased expression of survival factors (VEGF, pVEGFR2, Bcl-2, and pAkt, compared to WT SIGC, or those transfected with a scrambled RNAi sequence. * - denotes statistically different (p < 0.05) protein levels in CD36 knockdown cells compared to WT cells or those transfected with a scrambled sequence. **C**. ABT-898 significantly (p < 0.005) decreased SIGC proliferation and increased activated caspase-3 expression, while CD36 KD abrogated this effect. # - statistically different (p < 0.05) proliferation or apoptosis compared to the serum free (SF) treatment within that genotype; * statistically difference (p < 0.05) between WT and CD36 KD groups.

Plasmids were purified by the Qiagen plasmid purification mini kit. Plasmids were sequenced (Laboratory Services, University of Guelph) to confirm the sequence identity of the oligo. 1 × 10^5^/ml SIGC cells were cultured in DMEM\F12 (Gibco) supplemented with 10% FBS without antibiotic on 24 well plates. At approximately 70% confluence medium was removed, cells were washed in PBS, and 500 ul/well 1xOPTI-MEM reduced serum medium (Gibco) was added. Transfection was achieved using Lipofectamine2000 (Invitrogen) according to the manufacturer instructions. Stable cell lines were established from single-cell dilution colonies, plated in 10 ug/ml blasticidin (Invitrogen) for approximately 2 weeks to ensure elimination of any non-transfected cells. Stable cell line generation was confirmed with confocal microscopy to localize plasmid GFP within the SIGCs. To confirm knockdown, brightfield/darkfield immunofluorescence overlays with a phase-contrast inverted fluorescence microscope and Western blot analysis were performed on WT, Scrambled, and CD36KD SIGC in the log growth phase (Figure 1).

### Hormone assays, ovarian morphometry, and fertility

At the late antral/ovulatory phase of the ovarian cycle, WT and CD36-\- mice were killed by CO_2_ asphyxiation and blood was collected in a heparinized syringe via cardiac puncture and centrifuged at 1,000 × g for 10 min at 4°C. The plasma supernatant samples were collected and stored at −80°C until assayed. LH was assessed by using an ELISA kit from Endocrine Technologies (Newark, CA) (sensitivity, 0.5 ng/ml; intra-assay variation, 7%; inter-assay variation, 9.8%). FSH was measured by using an ELISA kit from Biocode-Hycel (sensitivity, 0.2 ng/ml; intra-assay variation, 4.7%; inter-assay variation, 8.5%). Ovaries were also collected, fixed in 10% neutral buffered formalin, processed, embedded in paraffin and 5 μm thick sections were cut throughout the entire tissue with a rotary microtome. For ovarian morphology assessment, every 5^th^ slide was subjected to Hematoxylin/Eosin staining and was used for counting of follicles and corpora lutea. Pre-antral/antral follicles and corpora lutea were counted in the 2 groups of mice (n = 6/group). As an index of fertility, ovarian synchronization was performed in a separate cohort of WT and CD36−/− null littermate mice (n = 6/group). Mice were injected with 2.5 IU PMSG, followed 48 hr later with 5 IU hCG (Sigma) to induce ovulation. Mice were immediately paired with proven male breeder mice and left together for one week before the male was removed. Mice were monitored for approximately 20 days following mating and the number of offspring from the female mice was counted.

### Cell proliferation and apoptosis

To identify the role of CD36 in TSP-1 mediated granulosa cell proliferation and apoptosis, SIGC were subjected to RNAi knockdown as described above. WT and CD36 knockdown (CD36 KD) cells were plated in 24 well plate on glass coverslips. At 60% confluence, medium was changed to reduced serum (1% FBS) DMEM/F12 medium (Gibco) overnight. Cultures were treated with 0, 20, 50 or 100 ng/ml of the TSP-1 mimetic peptide ABT-898 for 24 hours. Following treatment, cells were rinsed in PBS and fixed for 1 hour in 10% (vol/vol) neutral buffered formalin. Cells were then permeabililzed with 1% Triton X-100 (Sigma) in PBS for 15 min, followed by blocking in 5% BSA/0.1% Sodium Azide in PBS for 10 min. Cells were then either incubated overnight at 4°C with antibodies to phosphorylated Histone H3 antibody (proliferation; 1:2000 dilution; Abcam, ab5176) or anti-active Caspase-3 antibody (apoptosis; 1:500 dilution; Millipore, ab3623) followed by Alexa-Fluore 594-labeled donkey anti-rabbit secondary antibody (1:500 dilution, Invitrogen) for 1 hr at room temperature. After rinsing, cells were stained with 2ug/ml DAPI (Sigma) to counterstain nuclei blue and mounted on glass slides (SuperFrost Plus, Fisher) with Prolong Gold antifade solution (Invitrogen). Epifluorescence microscopy was used for image acquisition and integrated morphometry software (Metamorph, Burlingname, CA) was used to quantify the percent immunopositive cells in follicles without (pre-antral) or with (antral) an antrum, and in corpora lutea.

### Immunohistochemistry

Five micrometer-thick paraffin embedded ovarian tissue sections from wild-type and CD36^-\-^ mice were incubated overnight at 4°C in a humidified chamber with rabbit polyclonal anti-VEGF antibody (1:600 dilution; Santa Cruz Biotechnology, CA, sc152), rabbit polyclonal anti-VEGFR-2 antibody (1:200 dilution; Cell Signaling, 2479); goat polyclonal anti-TSP-1 antibody (1:600 dilution; Santa Cruz, sc59887); mouse monoclonal anti-CD31 (1:500 dilution; Abcam; ab28634); or mouse monoclonal anti-Ki67 antibody (1:500 dilution; Sigma, Oakville, ON, SAB4501880). The following day, biotinylated secondary antibody (1:100 dilution, Sigma) was applied for 2 hr at room temperature (RT), followed by horseradish peroxidase (Extravidin, 1:50 dilution, Sigma) for 1 hr at RT. Antigen localization was provided with incubation in DAB solution (SigmaFast 3,3′-Diaminobenzidine tablets), and tissues were counterstained with Carazzi’s Hematoxylin, dehydrated, cleared in xylene and mounted on coverslips. Images were captured using brightfield microscopy and the percentage of immunopositive tissue was quantified using a computer-generated thresholding algorithm and analysis (Aperio, ImageScope) for VEGF, VEGFR-2, and TSP-1 immunostaining. For CD31 staining, blood vessel density was calculated as the percentage of ovarian tissue comprised of CD31-postive endothelium using integrated morphometry software (Metamorph, CA). Ki67 staining in follicular and luteal cells were quantified manually by two independent observers blinded to whether the slides belonged to WT or CD36^−/−^ mice.

### Western blot analysis

Total cellular proteins were isolated from wild type and transfected SIGC cells and ovarian tissue from WT and CD36^−/−^ mice using standard RIPA buffer containing protease inhibitor cocktail. Denatured 20ug and 40ug protein were loaded to 4-15% gradient PAGE gel (Mini protean TGX Gel, BioRad) and transferred to polyvinylidene fluoride (PVDF) membrane (Millipore), blocked (5% skim milk in TBST) for 1 hour at room temperature. For in vitro experiments, primary antibodies with disparate molecular weights were incubated on the pvdf membrane that was cut around the area of the predicted molecular weight. Membranes were incubated with VEGF (1:500, Santa Cruz), TSP-1 (1:200, santa cruz), Bcl-2 (1:500, Novus Biological), VEGFR2 (1:2000, Cell Signaling), phospho VEGFR-2 (1: 500, Cell Signaling), CD36 (1:400, BD Pharmingen), Akt (1:100, Cell Signaling), phosphoAkt (1:500, Cell Signaling) and B-actin (1:4000, Cell Signaling) overnight at 4C on a rocking platform. Blots were washed in TBS with 1% Tween 20 (TBST) and incubated in appropriate dilutions of secondary antibodies (anti-rabbit IgG-HRP; anti-mouse IgG-HRP, Cell Signaling). Reactive protein was detected with Western Lightning Chemiluminescence Reagent Plus (PerkinElmer) on X-ray film (Kodak ClinicSelect blue). For some blots in which one of the primary antibodies was similar size to β-actin, the membrane was stripped using Millipore Re-Blot Plus MildTM (Millipore) for 15 min at RT, followed by 2 washes of 5% skim milk in TBST before re-probing with β-actin primary antibody and anti-rabbit secondary antibody. Films were imaged and densitometry analysis was performed using an AlphaInnotech imaging station.

### Statistical analysis

Three replicates of all data were performed and used to determine statistical significance. For immunohistochemistry experiments, a minimum of 5 fields of view at 200× magnification were used. Statistical analysis was performed using a one-way ANOVA, followed by Bonferonni’s post-hoc test. P values are listed in the figure legends.

## Results

### Knockdown of CD36 causes increased granulosa cell proliferation and survival

To evaluate the role of CD36 in granulosa cell function, we inhibited expression of the receptor in granulosa cells using RNA interference. With the RNA interference, we were able to reduce SIGC expression of CD36 by approximately 75% compared to WT cells or those transfected with scrambled oligonucleotide sequence (Figure 1A/B). Following knockdown, SIGCs had increased expression of the pro-angiogenic proteins VEGF and phosphorylated VEGFR2 (Figure 1B). SIGCs also exhibited an increase in protein levels of the cytoprotective proto-oncogene Bcl-2 and the pro-proliferative and pro-survival phosphorylated Akt (Figure 1B). In in vitro experiments the Thrombospondin-1 mimetic peptide ABT-898 caused a significant (p < 0.05) decrease in SIGC proliferation in WT cells, but there was no change in proliferation in SIGCs that had CD36 knocked down (Figure 1C). Conversely, ABT-898 induced SIGC apoptosis in WT cells, but this effect was abrogated with CD36 knockdown (Figure 1C).

### Ovarian morphometry, gonadotropin production and number of offspring are altered in CD36-\- mice

**Figure 2:**
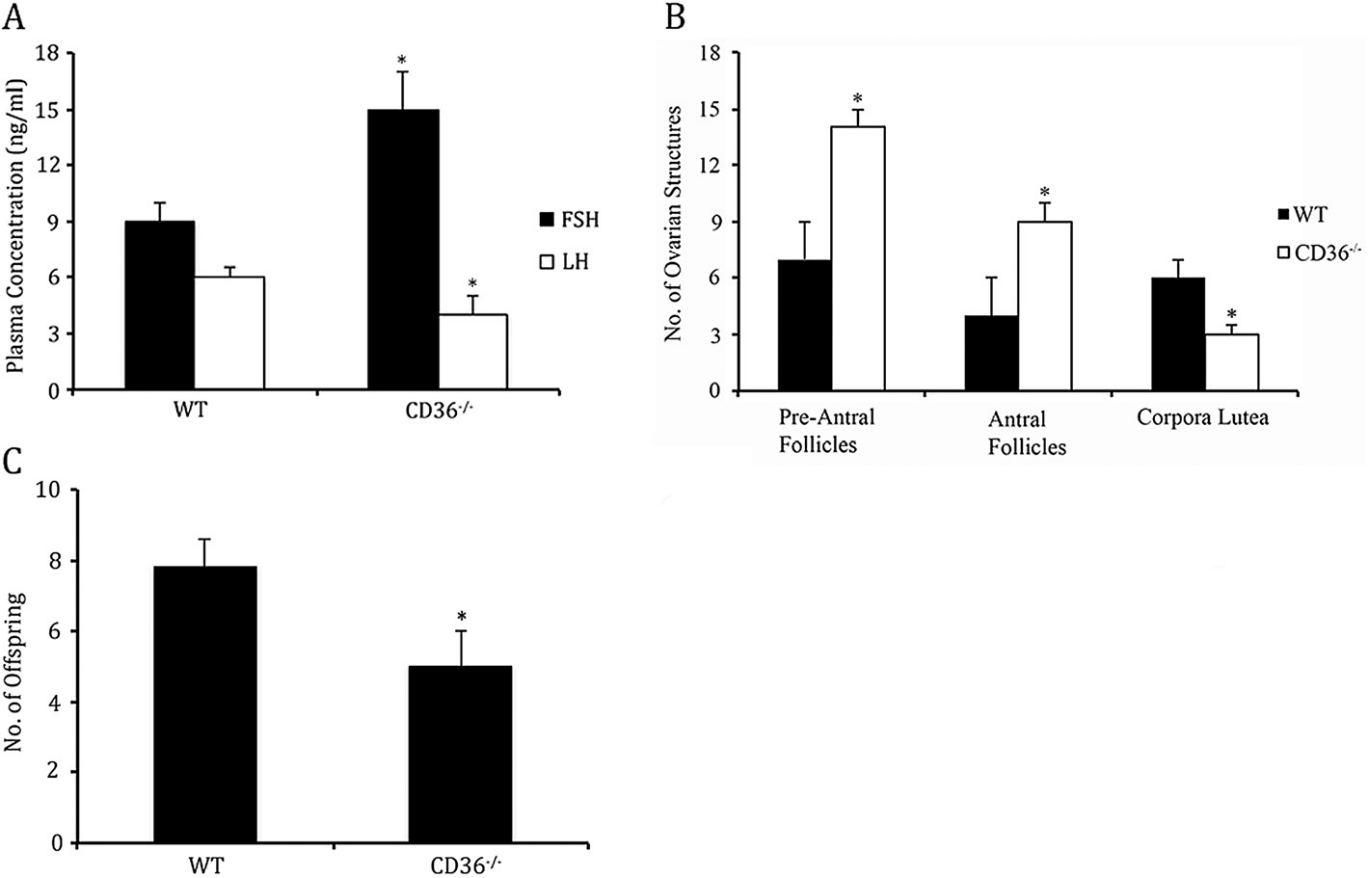
**Serum gonadotropin levels, ovarian morphology, and number of offspring in WT and CD36−/− mice. A**. ELISA was used to quantify changes FSH and LH in serum collected from CD36^−/−^ and WT mice. CD36^−/−^ mice had significantly (p < 0.05) higher circulating FSH and lower LH levels compared to WT controls. **B**. Morphometric analysis was conducted on histological sections from CD36^−/−^ and WT mice. Ovaries collected from CD36^−/−^ mice had significantly (p < 0.05) higher number of follicles and lower number of corpora lutei compared to WT controls. **C**. The number of offspring born to CD36−/− mice were fewer (p < 0.05) than WT control mice.

Serum samples from WT and CD36^-\-^ mice were collected at the time of euthanasia and subjected to ELISA analysis for leutenizing hormone (LH) and follicle stimulating hormone (FSH). FSH levels were significantly (p < 0.05) higher in CD36 null mice compared to WT controls (Figure 2A), while LH levels in circulation were significantly (p < 0.05) reduced compared to controls (Figure 2A). When ovarian structures were tabulated, there was a significant (p < 0.01) increase in the number of total follicles (including pre-antral, antral, and pre-ovulatory follicles) in CD36-\- mice compared to WT controls (Figure 2B). Conversely, CD36^-\-^ mouse ovaries contained significantly (p < 0.05) fewer corpora lutea than WT mouse ovaries (Figure 2B). The number of live offspring born from CD36^-\-^ mice was significantly (p < 0.05) lower than those from WT mothers (Figure 2C).

### Ovaries from CD36^-\-^ mice have higher expression of pro-angiogenic and pro-survival factors, compared to ovaries from WT controls

**Figure 3:**
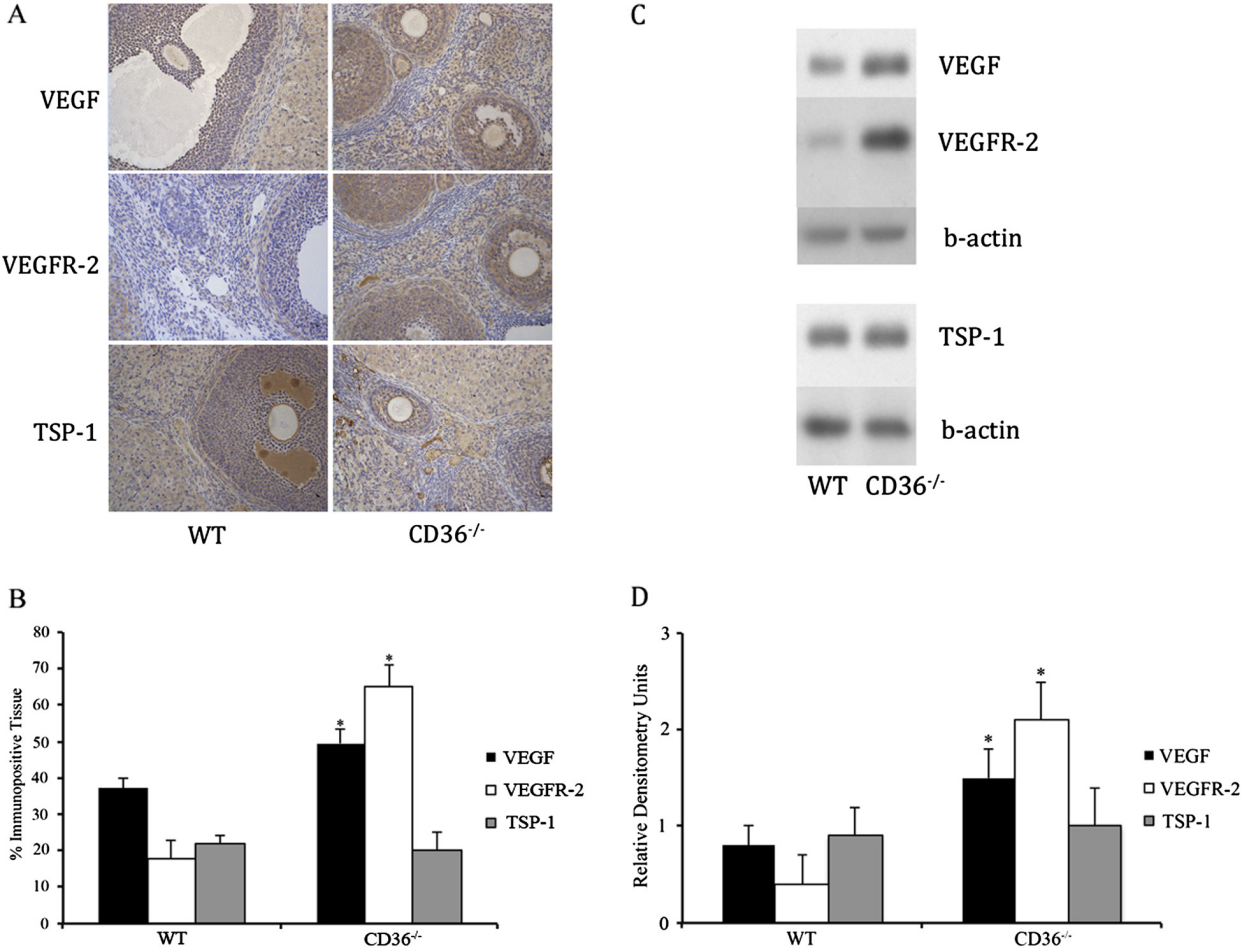
**Ovarian expression of VEGF, its receptor VEGFR-2, and TSP-1 in CD36−/− and WT mice. A**. Ovaries from CD36−/− and WT mice were immunostained for VEGF, VEGFR-2 and TSP-1. **B**. Immunohistochemical analysis showed that ovaries from CD36−/− mice had higher (p < 0.05) percentage positive ovarian tissue for VEGF and VEGFR-2 compared to WT controls. The percentage of tissue immunopositive for TSP-1 was not different between groups. **C**. Western blot analysis was performed on tissue lysates from WT and CD36−/− mice. **D**. There was a significant (* - p < 0.05) increase in VEGF and VEGFR-2 protein in CD36−/− ovaries compared to WT. No changes were observed in TSP-1 protein levels between groups.

Mice from both groups had their ovarian cycles synchronized and ovaries were collected at the late antral/pre-ovulatory phase of the cycle. Immunohistochemistry was performed to localize and quantify expression of pro-angiogenic and pro-survival VEGF and it’s receptor VEGFR-2, as well as TSP-1, which is the glycoprotein that binds and activates CD36. Ovaries from CD36^-\-^ mice had significantly (p < 0.05) higher expression of VEGF and VEGFR-2 (Figure 3), while levels of TSP-1 protein remained unchanged (Figure 3).

### CD36^-\-^ ovaries have increased proliferation and vascularity

**Figure 4:**
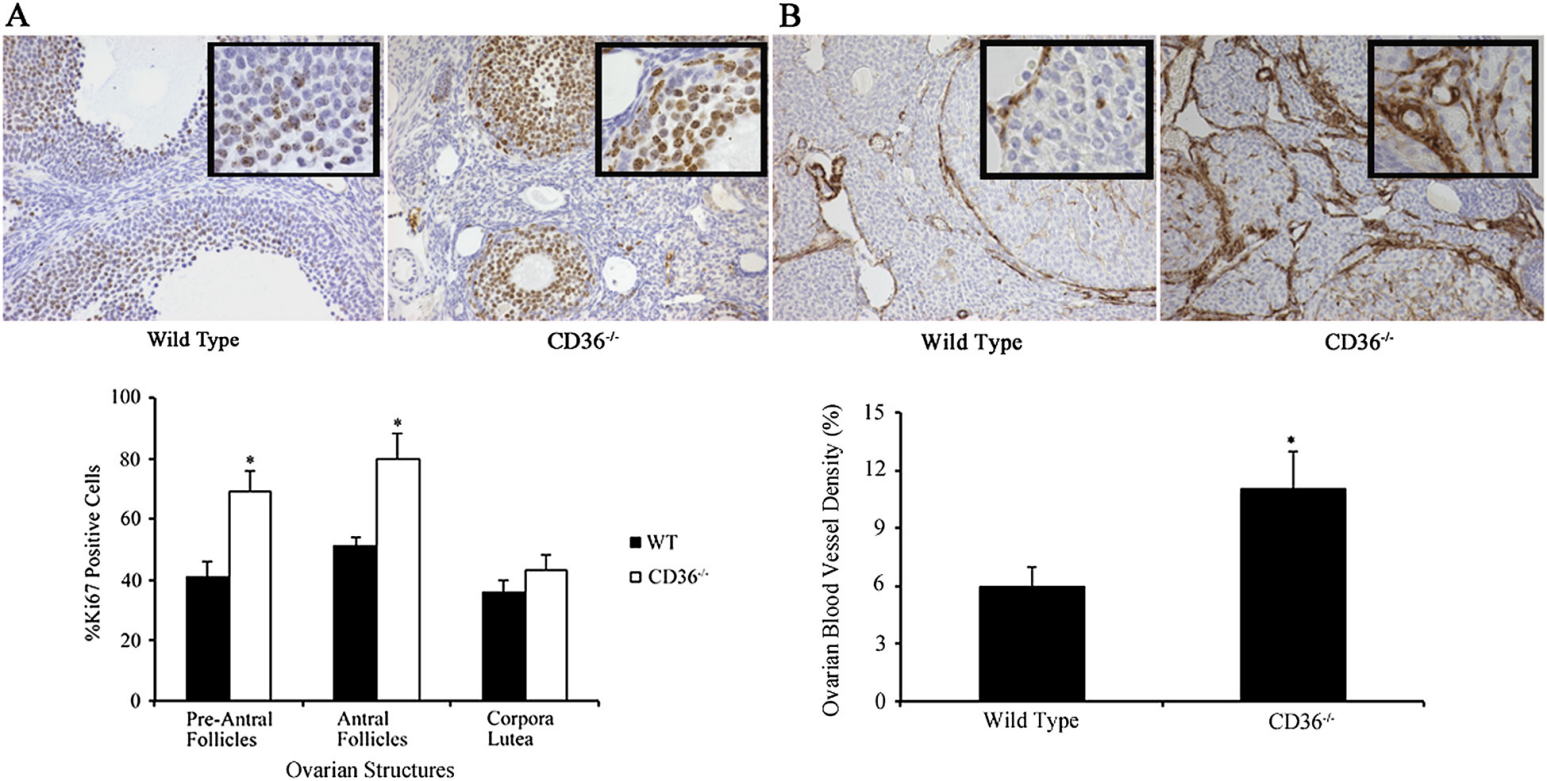
**Cell proliferation and blood vessel density in ovaries from CD36−/− and WT mice. A**. Immunohistochemistry for Ki67 showed that pre-antral and antral follicles from CD36−/− mice had a significant (p < 0.05) increase in the percentage of proliferating granulosa cells than WT controls. **B**. CD36−/− ovaries had a significantly (p < 0.05) higher blood vessel density than WT ovaries. Tissue sections are shown at 200× magnification; insets are at 600× magnification.

Ovaries from WT and CD36^-\-^ mice collected at the late antral/pre-ovulatory phase of the ovarian cycle were immunostained for Ki67 to quantify cell proliferation in follicular granulosa cells. Ovaries from CD36^−/−^ demonstrated a significant (p < 0.05) increase in granulosa cell proliferation in both pre-antral and antral-stage follicles (Figure 4A). Cellular proliferation in corpora lutea was not statistically different between groups (Figure 4A). Immunostaining with the endothelial cell marker CD31 was also performed to quantify changes in ovarian vascularity. Ovaries from CD36^-\-^ mice had a significant (p < 0.01) increase in microvessel density compared to WT control mice (Figure 4B).

## Discussion

This paper demonstrates the role of CD36 in the TSP-1 mediated effects in ovarian folliculogenesis. Based on the results of this study, CD36 appears to be an important regulator of ovarian angiogenesis, and follicular and luteal development. Disruption of expression of CD36 significantly alters ovarian morphology and expression of factors related to ovarian cell proliferation, survival, and angiogenesis. CD36 is expressed in granulosa and cumulus cells of rodents, bovines [16] and humans [27,28], although the specific function of the receptor is not completely understood.

In vitro, CD36 TSP-1 receptors are known to co-localize with VEGF receptors on cell membranes, and evidence suggests that these receptors may directly associate with each other and regulate signaling activity. We showed that knockdown of CD36 in ovarian granulosa cells resulted in an increase in expression of phosphorylated VEGFR2. The three type I repeat region of TSP-1 has been shown to reduce VEGFR2 phosphorylation and inhibit VEGF signal transduction [29] and the association between CD36, β1 integrins, and TSP-1 is thought to be important in mediating this inhibition [30]. In the CD36 null mice, there was a significant increase in ovarian blood vessel density compared to WT controls. In these mice, there was an increase in ovarian expression of VEGF and VEGFR2 and the in vitro data in this paper suggests that removal of the inhibitory influence of CD36 would allow for enhanced phosphorylation of VEGFR2, resulting in an increase in peri-follicular and luteal angiogenesis.

Knockdown of CD36 has been shown to increase proliferation and expression of survival and angiogenic in endothelial and tumor cells [25]. In this study, CD36 knockdown in vitro resulted in increased granulosa proliferation and survival, which was associated with increased granulosa cell expression of phosphorylated Akt. Akt phosphorylation is an important promoter of granulosa cell proliferation and viability, which are the driving forces behind follicle growth and maturation [31,32]. In vitro, the increased expression of phosphorylated Akt was associated with increased granulosa cell proliferation and decreased apoptosis. In vivo, CD36 null mice had increased granulosa cell proliferation and a greater number of growing follicles, compared to WT controls. Akt phosphorylation promotes follicle activation and survival [33] and inhibits follicle atresia [34,35]. Increased activity of the PI3K/Akt pathway in the absence of CD36 could also have contributed to the increased number of follicles present in the ovaries of the CD36 null mice. Thrombospondin signaling has been linked to follicle atresia previously [15,23,24,36] although the mechanisms have been unclear. This paper suggests that the PI3/Akt signaling pathway may be an important mediator of TSP-1’s effects in the ovary.

Normal function of the ovary is dependent on the tightly regulated angiogenic mechanisms that facilitate folliculogenesis, luteogenesis, and dissemination of the steroid hormones generated within the ovary. Angiogenesis within the ovary is a balance between and expression of pro- and anti-angiogenic factors. We have shown that members of the VEGF family and members of the anti-angiogenic TSP-1 are coordinately expressed during the ovarian cycle and have profound impacts on the angiogenic processes that occur throughout the cycle [15,16]. Within the ovary, cytokine action causes a downregulation of CD36, which is necessary to facilitate the explosive angiogenesis that occurs during luteal formation [37]. We showed in this study that CD36 null mice had significantly higher ovarian microvessel density compared to wildtype controls. CD36 appears to be critical in the regulation of physiological and pathophysiological angiogenesis. CD36 mediated TSP-1 signaling maintains corneal vascularity and CD36 deficiency leads to age-related corneal neovascularization while activation of CD36 can induce regression of inflammatory corneal angiogenesis [38]. CD36 has been implicated in the regulation of tumor angiogenesis. TSP-1, and other proteins containing the thrombospondin type-1 repeats (TSR), are known to endogenously inhibit the angiogenesis that occurs to facilitate tumor growth. CD36 has been shown to be required for the anti-angiogenic activity of these proteins and is absence impairs their angiogenesis inhibition and tumor vascularity increases and tumor growth is accelerated [17,39].

The morphological changes in the ovaries in the CD36 null mice somewhat replicate those seen in the condition of polycystic ovarian syndrome (PCOS) in which there is an increased number of primary follicles which remain preovulatory and do not progress to ovulation and formation of the corpus luteum [40]. In our study, CD36 null mice had an increased number of preovualtory follicles, concomitant with fewer corpora lutei, compared to WT controls. Elevated blood vessel density, and increased VEGF signaling were seen in the ovaries of the CD36 null mice and these are hallmarks of the pathogenesis of PCOS [41,42]. It has been shown that by increasing TSP-1 signaling, ovarian hypervascularization can be reversed as a method to treat PCOS [43]. Interestingly, CD36 null mice had elevated serum FSH, with suppressed LH, compared to WT controls. In classic PCOS, the hyperandrogenism and elevated GnRH generally results in an increase in the LH/FSH ratio [44]. In our mice, the elevated FSH may have been responsible for the increased number of recruited, but unovulated follicles as FSH is known to stimulate follicle development [45,46] and protect against follicle atresia [47,48]. FSH is a potent activator of the PI3K/akt signaling pathway [49] and the elevated circulating FSH seen in the CD36 null mice may have contributed to the increased akt phosphorylation observed in the ovaries from these mice. Reduced circulating LH in the CD36 null mice may have reduced the ovulatory stimulus, resulting in the increased number of pre-ovulatory follicles and fewer corpora lutei seen in these mice. CD36 has also been implicated in the pathogenesis of PCOS due to its role in regulating metabolism. CD36 is expressed in tissues regulated to fatty acid metabolism, including adipocytes [50]. Women with PCOS often exhibit higher levels of visceral obesity [51] and elevated CD36 expression in adipose tissue is seen in women with PCOS [11], further implicating the receptor in the pathogenesis of this disease. Levels of soluble CD36 are elevated in PCOS patients, and they are associated with the altered insulin sensitivity seen in this disease [52]. The metabolic profile of the CD36 null mice was not evaluated in this study, but will be the subject of investigation in the future. Transcriptome analysis has been performed on follicular cells from PCOS patients and healthy women. Haouzi et al. [53] showed that cumulus cells surrounding the oocyte had differential expression of a number of growth factors and genes involved in steroid metabolism. Granulosa cell expression of genes involved in inflammation, metabolism, and coagulation has also been implicated in the pathogenesis of PCOS [54,55]. After review of the gene lists published in the various transcriptome papers, CD36 was not specifically mentioned, although the cellular process that it is involved in have been implicated. A closer evaluation and comparison of CD36 expression in samples from PCOS patients and healthy women may be warranted.

## Conclusions

The results from this study suggest that TSP-1 and CD36 have important functions in the ovary and are potent regulators of follicular and luteal development, ovarian angiogenesis, and ovarian function. Disrupted expression of these proteins may be related to ovarian dysfunction and their roles in specific ovarian pathologies warrants further investigation.

## Competing interests

The authors declare that they have no competing interests.

## Authors’ contributions

JP initiated and designed the project, performed data analysis and wrote the manuscript. KO performed the in vitro CD36 knockdown experiments and MR performed the immunohistochemistry and Western blot analysis. All authors read and approved the final manuscript version.
